# Prospects for Controlling Hepatitis B Globally

**DOI:** 10.3390/pathogens13040291

**Published:** 2024-03-29

**Authors:** Vicente Soriano, Víctor Moreno-Torres, Ana Treviño, Fernando de Jesús, Octavio Corral, Carmen de Mendoza

**Affiliations:** 1 UNIR Health Sciences School & Medical Center, 28010 Madrid, Spain; 2 Department of Internal Medicine, Puerta de Hierro University Hospital, Majadahonda, 28222 Madrid, Spain

**Keywords:** chronic hepatitis B, tenofovir, bulevirtide, entecavir, hepatitis delta, long-acting antivirals, gene editing

## Abstract

Infection with the hepatitis B virus (HBV) is highly prevalent globally. Over 250 million people suffer from chronic hepatitis B, and more than 800,000 patients die each year due to hepatitis B complications, including liver cancer. Although protective HBV vaccines are recommended for all newborns, global coverage is suboptimal. In adults, sexual transmission is by far the most frequent route of contagion. The WHO estimates that 1.5 million new HBV infections occur annually. Oral nucleos(t)ide analogues entecavir and tenofovir are the most frequent antivirals prescribed as HBV therapy. Almost all patients adherent to the medication achieve undetectable plasma viremia beyond 6 months of monotherapy. However, less than 5% achieve anti-HBs seroconversion, and viral rebound occurs following drug discontinuation. Therefore, nucleos(t)ide analogues need to be lifelong. New long-acting formulations of tenofovir and entecavir are being developed that will maximize treatment benefit and overcome adherence barriers. Furthermore, new antiviral agents are in development, including entry inhibitors, capside assembly modulators, and RNA interference molecules. The use of combination therapy pursues a functional HBV cure, meaning it is negative for both circulating HBV-DNA and HBsAg. Even when this goal is achieved, the cccDNA reservoir within infected hepatocytes remains a signal of past infection, and HBV can reactivate under immune suppression. Therefore, new gene therapies, including gene editing, are eagerly being pursued to silence or definitively disrupt HBV genomes within infected hepatocytes and, in this way, ultimately cure hepatitis B. At this time, three actions can be taken to push HBV eradication globally: (1) expand universal newborn HBV vaccination; (2) perform once-in-life testing of all adults to identify susceptible HBV persons that could be vaccinated (or re-vaccinated) and unveil asymptomatic carriers that could benefit from treatment; and (3) provide earlier antiviral therapy to chronic HBV carriers, as being aviremic reduces the risk of both clinical progression and transmission.

## 1. Introduction

**Figure 1:**
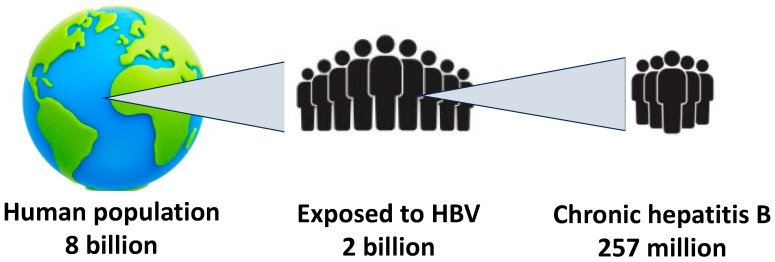
The global burden of hepatitis B.

Infection with the hepatitis B virus (HBV) is highly prevalent worldwide. Roughly two billion people have been exposed to HBV globally (Figure 1). Although acute HBV infection is controlled by the majority of adults, over 250 million people suffer from chronic hepatitis B [1], and more than 800,000 patients die each year due to hepatitis B complications, including liver cancer [2].

**Figure 2:**
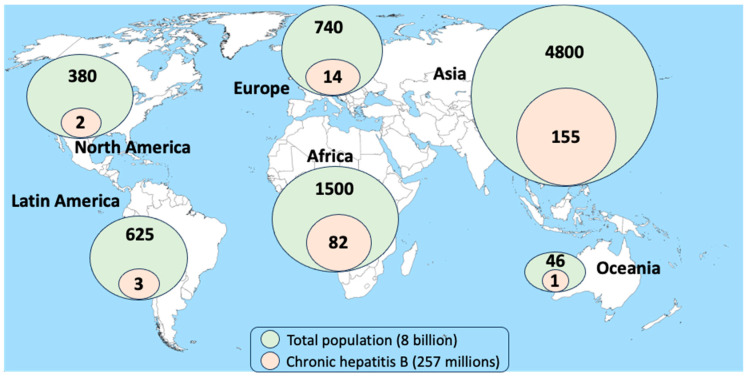
Geographic distribution of chronic hepatitis B.

Although protective HBV vaccines have existed for more than 40 years and HBV vaccination is recommended for all newborns, global coverage is suboptimal, especially in poor-resource countries [3]. Asia and Africa are the regions with the largest number of patients with chronic hepatitis B (Figure 2). Approximately a half live in three countries: China, India, and Indonesia [1]. In Western countries, migration flows from HBV-endemic regions largely account for most new chronic hepatitis B diagnoses [2,3]. In addition, HBV immunity waning might be more common than expected and explain the high rate of HBV-susceptible adults in countries with proper HBV vaccination policies [4]. The WHO estimates that 1.5 million new HBV infections occur annually worldwide [2].

Progression to chronicity is frequent in newborns following HBV exposure from infected mothers [5]. In contrast, chronic hepatitis B only develops in less than 5% of adults first exposed to the virus [5]. By far, sexual transmission is the most frequent route of contagion in adults. Transfusions and injection drug use are other major sources of HBV infection [5].

In the absence of treatment, the lifetime risk of developing cirrhosis or liver cancer is 30–40% in patients with chronic hepatitis B [1]. Interferon alfa was the first drug used as HBV therapy, but its limited efficacy and frequent side effects have precluded its wider use. Nowadays, the oral nucleos(t)ide analogues entecavir and tenofovir are the most frequently prescribed antivirals for HBV therapy [6]. Almost all treated and adherent patients achieve undetectable plasma viremia beyond 6 months of monotherapy. However, less than 5% achieve anti-HBs seroconversion, and viral rebound is the rule following drug discontinuation. Hence, nucleos(t)ide analogues need to be lifelong [6,7].

**Figure 3:**
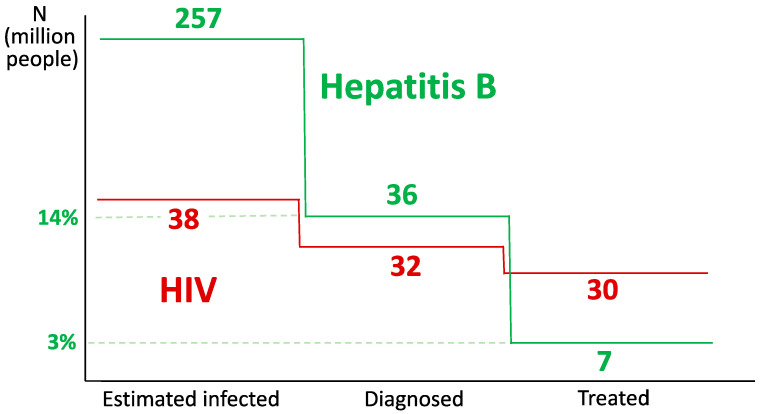
Comparison of the cascade of care for hepatitis B and HIV/AIDS.

The WHO set up in 2016 a plan to eliminate viral hepatitis as a major public health threat by 2030, proposing specific targets that include a 90% reduction in new diagnoses of chronic hepatitis B, 80% antiviral treatment coverage, and a 65% reduction in mortality due to HBV [8]. Up to 2024, only 36 (~14%) out of the 257 million people estimated to live with chronic hepatitis B have been diagnosed. Furthermore, only 7 million (~3%) are under antiviral treatment, and it is unknown whether this proportion is virally suppressed. Thus, the cascade of care for HBV is clearly behind the one for HIV/AIDS (Figure 3), for which the WHO has set up 95-95-95 goals for diagnosis, treatment, and suppression by 2030 [8].

New long-acting formulations of tenofovir have been developed that maximize treatment adherence. Furthermore, new antiviral agents are in development [7,9] to be used as combination therapy in an attempt to reach a functional HBV cure, meaning they are negative for both circulating HBV-DNA and HBsAg. Even when this goal is achieved, the cccDNA reservoir within infected hepatocytes remains a signal of past HBV infection, and reactivation may occur under immunosuppression [10]. New gene therapies, including gene editing, are eagerly being pursued to definitively silence or disrupt HBV genomes within infected hepatocytes and, in this way, ultimately cure hepatitis B [11].

## 2. New HBV Vaccines and Policies

Protective HBV vaccines were first introduced in the 1980s. Although the WHO recommends universal HBV vaccination for newborns, 15% do not receive it [3]. In addition, suboptimal responses and HBV immunity waning might be more common than expected [4].

Antiviral prophylaxis with nucleos(t)ide analogues during the third trimester of pregnancy in HBsAg+ mothers with high viral loads is complementary to HBV vaccination of newborns to prevent mother-to-child transmission.

The effectiveness of the HBV vaccine is determined by measuring serum anti-HBs. Values > 10 mIU/mL are considered protective. A recent meta-analysis showed that HBV seroprotective levels did not reach 60% in African children that had been vaccinated earlier in life [12]. This is a big threat to achieving the global hepatitis B elimination goal by 2030. Newer HBV vaccines are more immunogenic and would increase protection [13].

Recently, the CDC recommended one-time universal HBV screening of all adults [14], in an attempt to identify susceptible individuals that will benefit from vaccination and increase the proportion of people living with hepatitis B who are aware of their infection to 90% by 2030. It should be noted that chronic hepatitis B behaves as a silent killer because many carriers have no symptoms and are not aware of their infection. Furthermore, they can transmit the virus inadvertently through sex and unsafe injections, as well as during pregnancy. Overall, universal one-time HBV screening would be highly cost-effective [14].

Universal one-time HBV screening of adults should include the triple panel of HBsAg, anti-HBsAg, and anti-HBc. Each of these markers, respectively, would identify patients with active infection, immunity, and resolved infection that could be reactivated. People with negative results for all three tests are susceptible to HBV infection and should be vaccinated [15]. Awareness of whether the patient has resolved or depicts an active infection is crucial because many newer immunotherapies may potentially cause HBV reactivation [16]. Interestingly, the new CDC recommendation does not require adults to receive pre-vaccination hepatitis B testing. However, when feasible, the triple panel could save vaccination costs.

## 3. Current Hepatitis B Antivirals

Antiviral agents to treat HBV have been available for decades. Although viral suppression is achieved in most hepatitis B patients treated with either tenofovir or entecavir, HBV is not eradicated from carriers, and therefore oral treatment needs to be lifelong. Until recently, oral HBV treatment has been recommended for those with elevated liver enzymes, advanced liver fibrosis, and/or serum HBV-DNA > 2000 IU/mL.

Patients with chronic hepatitis B under long-term tenofovir benefit from a reduced risk of liver disease progression [17,18] and of hepatocellular carcinoma [19,20]. However, the risk of HCC still persists in cirrhotics. Moreover, the benefits of HBV suppression for those with low serum HBV-DNA remain largely unknown.

New HBV biomarkers, including quantitative HBsAg (qHBsAg), serum HBV-RNA, and HBV core-related antigen (HBcrAg), may help to improve our understanding of the natural history of chronic hepatitis B and its response to antiviral therapy.

Viral suppression is additionally associated with halted HBV transmission, a benefit known as ‘treatment as prevention’, given that undetectable viremia translates into a lack of contagiousness. This phenomenon was first described in the HIV field using antiretrovirals [21], but similarly applies to hepatitis B. Indeed, ‘test and treat’ strategies are being advocated for hepatitis B, acknowledging that they will be cost-effective [22].

## 4. New HBV Antivirals

**Figure 4:**
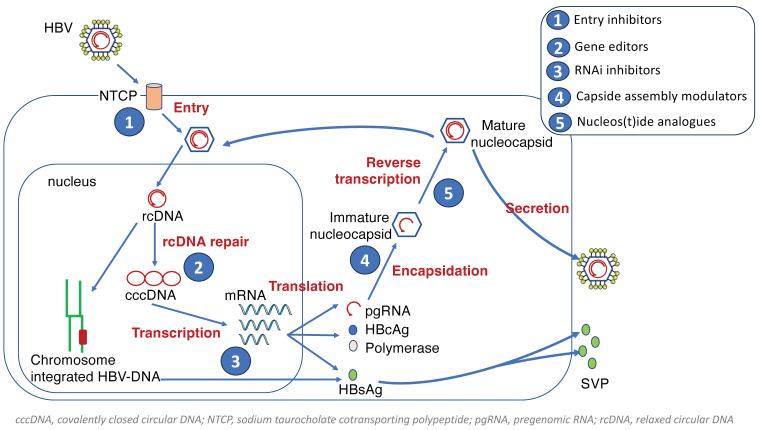
HBV life cycle (modified from [6]).

Novel therapies for chronic hepatitis B are in clinical development. New agents are aimed at inhibiting viral replication, reducing antigen production, and/or restoring host immune control [23]. The most advanced antiviral classes for hepatitis B are entry inhibitors, capsid assembly modulators, RNA interference molecules, and immunomodulators. Figure 4 records graphically the hepatitis B life cycle and the targeted steps with antivirals.

Bulevirtide is an entry inhibitor for hepatitis B and D viruses. It blocks the NTCP receptor at the hepatocyte surface, causing inhibitory competition with the physiologic substrate, the bile acids. In several clinical trials, bulevirtide has demonstrated excellent tolerability and minimal side effects [24]. Serum HBV-DNA goes down in most treated patients; however, no effect is seen on serum HBsAg concentrations. Bulevirtide is being tested in combination with other antivirals as HBV therapy. The drug has already been approved as a treatment for hepatitis delta.

Capsid assembly modulators inhibit HBV replication by targeting encapsidation of the pregenomic viral RNA and blocking early viral life cycle stages, including cccDNA formation. Two molecules, GLS4 and NJ-56136379, are completing phase II clinical trials [6,7,23].

Immunomodulators such as TLR agonists, monoclonal antibodies, checkpoint inhibitors, and therapeutic vaccines could enhance HBV-specific immune responses. However, TLR-8 agonists from Gilead’s selgantolimob and vesatolimob have failed to demonstrate any efficacy. Furthermore, the overall results with other immunotherapies have been quite disappointing to date [23].

## 5. Pre-Exposure Prophylaxis for Hepatitis B

A protective effect of tenofovir on HBV acquisition was originally reported in studies conducted in HIV-infected patients on antiretroviral therapy [25]. Sexually active men having sex with men (MSM) that received tenofovir as part of their antiretroviral regimen had a lower chance of HBV acquisition than those that did not receive the drug [26,27]. More recently, this protective effect on hepatitis B was also confirmed in HIV-uninfected persons at risk undergoing HIV pre-exposure prophylaxis (PrEP) with tenofovir. Based on these findings, the recent increasing trend in HIV therapeutics for using mono or dual regimens sparing tenofovir is worrisome. It could be associated with a rebound of incident HBV infections [28].

Although HBV vaccines are effective, individuals with immunosuppression may experience more frequent suboptimal responses and/or waning of antibodies following immunization [29]. Moreover, the circulation of HBV vaccine escape mutants has been shown to be responsible for anecdotal cases of acute HBV infection in vaccinated individuals [30]. For all these considerations, the use of tenofovir as HBV chemoprophylaxis might be considered in certain populations to avert breakthrough HBV infections. This could be the case of immunodeficient individuals engaged in high-risk behaviors, including injection drug users that share needles, MSM, and other persons with multiple sex partners. An additional indirect benefit of using tenofovir chemoprophylaxis would derive from reducing the chances of hepatitis delta virus (HDV) acquisition, since this virus requires HBV for transmission.

Entecavir has also been used for HBV chemoprophylaxis. It has a safer kidney profile than tenofovir; however, it has a lower resistance barrier, which can be risky for poor drug-adherent individuals. Entecavir has been used in adherent patients with autoimmune diseases receiving rituximab, biological agents, cyclophosphamide, or high-dose steroids, as well as in transplant patients under immunosuppressors. Rather than for PrEP, entecavir could be prioritized for preventing HBV reactivation under immunosuppression when renal toxicity is an important caveat [16].

## 6. HBV Gene Therapies

Several strategies are being attempted to stall HBV gene expression through HBV mRNA destabilization/inhibition, host protein modulation, or direct cccDNA mutagenesis. RNA interference (RNAi) molecules are used to suppress the post-transcriptional functions of HBV RNA transcripts (Figure 4). Briefly, small interference RNA (siRNA) molecules or antisense oligonucleotides (ASO) are designed to contain HBV-specific complementary sequences that either destabilize or facilitate the degradation of viral transcripts upon hybridization [31].

Several RNAi molecules have been developed and modified for improved intracellular delivery, stability, and function. AB-729 is a safe and well-tolerated suppressor of HBsAg in clinical development. RG-6346 and JNJ-3989 have proven effective in HBsAg reduction during early-phase clinical trials. ALG-125755 is another RNAi agent with higher stability and efficacy [32].

Bepirovirsen is an ASO that targets all HBV mRNAs and acts by decreasing levels of viral proteins. In a phase 2b trial that included 457 HBsAg+ patients, bepirovirsen at a dose of 300 mg injection per week for 24 weeks resulted in sustained HBsAg and HBV DNA loss in 10% of participants with chronic HBV infection [33]. However, RNAi agents only transiently silence gene expression and do not provide a complete deactivation of the persistent cccDNA.

## 7. Gene Editing for Hepatitis B

HBV cure strategies can be categorized into the following two types: functional cures and sterile cures. A functional cure for HBV involves the loss of HBsAg, with or without the development of anti-HBs, as well as undetectable serum HBV-DNA. The persistence of cccDNA with low or no transcriptional activity will allow treatment to be stopped, as the activated immune system should be able to control the remaining pool of HBV-infected cells.

Because HBsAg secretion in chronic HBV infection largely originates from HBV DNA integrated in the host genome, mutagenizing the highly conserved domains in the S gene could potentially suppress the expression of HBsAg. In this regard, cytosine base editors have demonstrated their efficacy [34,35].

In the path for HBV sterile cure, the cccDNA is completely destroyed or eliminated. This molecule is a fairly small (3.2 kb) compact genome containing tightly overlapped open reading frames (ORFs) encoding the genes necessary for viral survival. These unique features, in addition to its few copy numbers per infected cell, make cccDNA a potential substrate for gene editing. CRISPR-based systems use engineered RNA-guided Cas nucleases to generate site-specific insertion-deletion (indel) mutations. Their role as anti-HBV agents is eagerly being pursued [34,36,37].

## 8. Long-Acting Antiviral Formulations for Hepatitis B

The advent of long-acting formulations of tenofovir [38,39,40] would represent an attractive alternative option for both treatment and prophylaxis of hepatitis B. Long-term adherence to medications is generally challenging, as many patients tend to forgive daily dosing. The prescription of medications that only need to be given every two months or even with longer lags between doses has already demonstrated its benefit in the HIV field. A similar scenario would be expected with respect to hepatitis B [41].

Long-acting formulations of entecavir have also been developed [42]. Given the concern about the long-term side effects on kidneys and bones, extended-released entecavir medications might provide a good alternative option for a subset of patients.

## 9. Occult Hepatitis B

A subset of individuals exposed in the past to HBV depict sustained low-level serum HBV-DNA in the absence of positive HBsAg. As expected, most of them are positive for anti-HBc. Occult B infection (OBI) has clinical relevance in at least four scenarios: (1) HBV can be transmitted, causing a classic form of hepatitis B, through blood transfusion or liver transplantation; (2) it may reactivate in the case of immunosuppression, leading to hepatitis flares or even fulminant hepatitis; (3) it may accelerate the progression toward cirrhosis caused by other etiologies; and (4) it keeps the risk of developing hepatocellular carcinoma [43].

Nucleic acid testing (NAT), as performed in transfusion centers, may allow for the identification of OBI individuals, but low-level viremia can occasionally be missed [44]. Serum anti-HBc is generally positive but has low specificity for detecting OBI. In other words, it may produce many false positives for OBI [45].

In the HIV population, the rate of OBI was presumed to be greater, but nowadays, for most patients on antiretroviral therapy, including anti-HBV agents (i.e., tenofovir, emtricitabine, or lamivudine), this is no longer a concern [46]. However, the broader use of new tenofovir-sparing antiretroviral regimens (i.e., dolutegravir-rilpivirine) might account for a resurgence of OBI in the HIV setting [28].

## 10. Hepatitis Delta Superinfection

HDV is a unique, small, defective virus that requires HBV to complete its replication cycle. The 1700 single-stranded circular RNA genomes and a single antigen use the HBV envelope to form the HDV particle [47,48].

Roughly 5% of individuals with chronic hepatitis B are superinfected with HDV. Of the estimated 5 million HDV carriers worldwide, China, Pakistan, and Mongolia are the three countries with the largest number of individuals with chronic hepatitis delta [49]. Hot spots have been noticed in West-Central Africa, Mongolia, Taiwan, Pakistan, Turkey, and the Amazon basin [50]. In addition, HDV has spread globally among injection drug users [51]. This explains the disproportionately high prevalence of triple HIV-HBV-HDV coinfection [52].

Hepatitis delta is the most severe form of chronic viral hepatitis, leading to cirrhosis and liver cancer in more than half of infected patients [53,54]. Until recently, only peginterferon-alfa was used to treat hepatitis delta. However, this drug is not well tolerated, and only less than 30% of patients respond to therapy. Furthermore, a large proportion relapse upon drug discontinuation [55].

Bulevirtide is a new entry inhibitor that blocks the interaction of hepatitis B and D with the NTCP receptor at the hepatocyte surface [24]. As a defective virus, HDV uses HBsAg as part of its envelope and, in this way, enters hepatocytes using the same receptor as HBV. Both clinical trials and real-world experience have demonstrated the virological efficacy and safety of bulevirtide in patients with chronic hepatitis delta [56,57].

Phase 3 trials with lonafarnib as HDV therapy are ongoing [58]. This drug is already approved to treat progeria, a rare genetic disease. In hepatitis delta, lonafarnib specifically blocks the assembly of virions within hepatocytes. The development of lonafarnib, however, has recently been halted due to unexpectedly serious side effects. Peginterferon lambda is another antiviral drug currently being tested in phase 3 studies as a hepatitis D treatment [59].

Since there is no stable cell reservoir for the HDV-RNA genome, viral clearance might hypothetically be achieved if complete blocking of viral replication occurs using antivirals for a minimum timeframe [60]. The combination of several specific anti-HDV agents will be required. This is what happens in hepatitis C, combining direct-acting antivirals that cure nearly all patients treated for 2–3 months. Hepatitis delta is a unique condition, and clearance of HDV-RNA genomes might occur despite HBV persistence as cccDNA or integrated HBV-DNA within hepatocytes. Supporting this concept are cases of HDV elimination despite persistence of serum HBsAg following treatment with bulevirtide or lonafarnib plus peginterferon [61,62]. The advent of long-acting formulations for these drugs might hypothetically open the opportunity for considering the cure of hepatitis delta, perhaps using single shots [60].

## 11. Conclusions

The global burden of chronic hepatitis B is huge and will continue to increase since the risk of clinical complications, including liver decompensation and hepatocellular carcinoma, is age-dependent and the world population is aging [63,64,65]. In Western countries, despite universal HBV vaccination and easy access to oral antivirals, the waning of HBV antibodies and migration flows from HBV-endemic regions account for sustained rates of new HBV infections. There is a need to unveil the mechanisms of anti-HBs antibody waning, and periodic surveys of immunized populations and re-vaccination of certain high-risk groups should be considered.

New antiviral agents are in development to be used as combination therapy in an attempt to reach a functional HBV cure, meaning negativity of both circulating HBV-DNA and HBsAg. Even when this goal is achieved, the cccDNA reservoir within infected hepatocytes remains a signal of past HBV infection, and under immune suppression, HBV can reactivate.

New gene therapies, including gene editing technologies, are eagerly being pursued to definitively disrupt HBV genomes within infected hepatocytes and, in this way, ultimately cure hepatitis B. In the meantime, three actions can be taken to push HBV eradication globally: (1) expand universal HBV vaccination of newborns; (2) perform once-in-life testing of all adults to identify susceptible HBV persons that could be vaccinated (or re-vaccinated) as well as unveil asymptomatic HBsAg carriers that could be treated; and (3) promote earlier and broader antiviral treatment of chronic HBV carriers to maximize the proportion of them being aviremic [66]. At this time, less than 3% of chronic hepatitis B patients are treated worldwide. Ultimately, expanding HBV treatment to all viremic individuals will be cost-effective [67]. HIV-HBV-HCV test and treatment strategies have been proposed as the most effective path to achieve the WHO goals for 2020 [22].

Being aviremic is important since there is a double benefit of suppressing viral replication in HBsAg+ individuals (Table 1). On one hand, the patient would reduce the risk of: (i) liver fibrosis progression [17,18]; (ii) developing hepatocellular carcinoma [19,20]; (iii) experiencing HBV reactivation under immunosuppression; and (iv) manifesting extrahepatic complications. On the other hand, it halts the risk of transmission to others, following the U = U paradigm (undetectability means untransmissibility), clearly established in the HIV field [21].

## Figures and Tables

**Table 1:** Benefits of expanding HBV therapy to all viremic patients.

For the individual:
Lower risk of liver fibrosis progression Reduced risk of developing liver cancer Less likelihood of experiencing HBV reactivation under immunosuppression Less risk of developing extrahepatic manifestations
For the society:
Minimal risk of transmission to others (sex, nosocomial, mother-to-child, etc.)

## Data Availability

Not applicable.
